# Chronic Intermittent Hypoxia Regulates CaMKII-Dependent MAPK Signaling to Promote the Initiation of Abdominal Aortic Aneurysm

**DOI:** 10.1155/2021/2502324

**Published:** 2021-12-21

**Authors:** Chenyu Xu, Jun Xu, Chunfang Zou, Qian Li, Shan Mao, Ying Shi, Yan Tan, Wei Gu, Liang Ye

**Affiliations:** Department of Respiration, Nanjing First Hospital, Nanjing Medical University, Nanjing 210006, China

## Abstract

Obstructive sleep apnea (OSA) is highly prevalent in patients with abdominal aortic aneurysm (AAA). However, the effects of OSA on AAA initiation in a murine model of sleep apnea have not been completely studied. In this paper, Apoe^−/−^ C57BL/6 mice infused with angiotensin II (Ang II) were placed in chronic intermittent hypoxia (CIH) condition for inducing OSA-related AAA. CIH significantly promoted the incidence of AAA and inhibited the survival of mice. By performing ultrasonography and elastic Van Gieson staining, CIH was found to be effective in promoting aortic dilation and elastin degradation. Immunohistochemical and zymography results show that CIH upregulated the expression and activity of MMP2 and MMP9 and upregulated MCP1 expression while downregulating *α*-SMA expression. Also, CIH exposure promoted ROS generation, apoptosis, and mitochondria damage in vascular smooth muscle cells (VSMCs), which were measured by ROS assay, TUNEL staining, and transmission electron microscopy. The result of RNA sequencing of mouse aortas displayed that 232 mRNAs were differently expressed between Ang II and Ang II+CIH groups, and CaMKII-dependent p38/Jnk was confirmed as one downstream signaling of CIH. CaMKII-IN-1, an inhibitor of CaMKII, eliminated the effects of CIH on the loss of primary VSMCs. To conclude, a mouse model of OSA-related AAA, which contains the phenotypes of both AAA and OSA, was established in this study. We suggested CIH as a risk factor of AAA initiation through CaMKII-dependent MAPK signaling.

## 1. Introduction

Obstructive sleep apnea (OSA) refers to the repeated upper airway obstruction during sleep, which can cause hypopnea and apnea. The main clinical features of OSA include snoring, frequent awakening, witnessed apneas, recurrent hypoxemia, hypercapnia, and sleep fragmentation, which seriously affect the quality of life of patients with OSA. It has been reported by the Wisconsin Sleep Cohort study that the prevalence of moderate to severe OSA among 30-70 years old adults seems to be 10% [1]. Increasing studies have confirmed OSA as one risk factor of hypertension, coronary artery disease, stroke [2–4], and various cancers, like lung, head, and neck cancers [5, 6]. Of note, OSA is highly prevalent in patients with abdominal aortic aneurysm (AAA) [7], and severe OSA may promote the expansion of AAA [8].

AAA is the irreversible dilation of abdominal aorta. It can be defined if the abdominal aorta dilates to 1.5 times the diameter of the adjacent vessels or the aorta size is greater than 30 mm [9]. AAA is usually asymptomatic unless it ruptures. Once ruptured, the mortality is higher than 85% [10]. Despite the development of treatment strategy, no effective drug therapies have been used in clinic due to the limited understanding of AAA pathogenesis. Further investigations focused on the initiation of AAA are urgently needed. Patients with OSA may be prone to the initiation of AAA since the repeated apnea leads to sympathetic nerve activation, arterial pressure elevation, oxidative stress, and excessive inflammation [8, 10]. However, the effect of OSA on the initiation of AAA has not been completely studied.

Chronic intermittent hypoxia (CIH) is one of the main features of OSA. Since CIH is considered to be the main pathological cause of OSA-related cardiovascular diseases, CIH model has been widely used for the basic studies [11]. Under hypoxia, cells adapt to the anoxic conditions by automatic regulation, while in the reoxygenation stage, the sharply increased oxygen in cell leads to the excessive production of reactive oxygen species (ROS) and cell death [12]. Oxidative stress and inflammation has been considered two key pathological processes in the initiation of AAA. However, the effects of CIH on AAA are largely unknown. In this study, we firstly established a mouse model of OSA-related AAA, which contains the phenotypes of both AAA and OSA. Moreover, the influence of OSA on the initiation of AAA as well as the underlying mechanism was studied.

## 2. Materials and Methods

### 2.1. Animals

Male Apoe^−/−^ C57BL/6 mice aged 8 weeks were obtained from Model Animal Research Center of Nanjing University (Nanjing, China). The animal studies were approved by the Animal Ethics Committee of Nanjing Medical University and performed strictly according to the Guideline of AAALAC International. Mice were placed in standard breeding cages provided with standard mouse chow and water, as previously described [13].

### 2.2. AAA Mouse Model and CIH Protocol

Apoe^−/−^ mice were implanted with an Alzet model 2004 miniosmotic pumps (DURECT, USA), through which 1 × 10^3^ ng/min/kg angiotensin II (Ang II, Sigma-Aldrich, St. Louis, MO, USA) was infused for four consecutive weeks. The mice infused with the same volume of saline through the pumps were considered the negative control. After infusion, mice were placed in an Animal Hypoxic Chamber (ProOx-100, Tow-Int Tech, Shanghai, China), of which the N_2_ and O_2_ contents can be controlled by computer. The CIH profile designed to produce similar nadir hemoglobin oxygen saturations and apnea/hypopnea index as observed in severe OSA patients. During CIH exposure, the chamber was flushed with 100% N_2_ to reduce the inspired O_2_ to 9% and this procedure lasted for 60 sec, after which the inspired O_2_ return to the 21%. The cycle was repeated every 2 min for 8 h/day, 7 days/week for four consecutive weeks. The mice in the control group were treated the same as the CIH group, but room air was used to flush chamber instead of 100% N_2_. Four weeks later, mice were sacrificed and the abdominal aortas from mice were collected for photographs taken and the following histological analyses.

### 2.3. Abdominal Ultrasonography

Abdominal ultrasonography for mice was conducted under the anaesthetization of 1.5% isoflurane. Abdominal hairs were shaved off, and abdominal echography was performed by using Vevo 2100 ultrasound (VisualSonics, Toronto, Canada) with a 30 MHz transducer. The suprarenal abdominal aortic diameter was measured by a real-time microvisualization scan head (RMV 704, Visual Sonics) with a central frequency of 40 MHz.

### 2.4. RNA Sequencing and Bioinformatics Analysis

The suprarenal abdominal aortic tissues were collected from mice treated by Ang II together with CIH or ambient 21% O2 (normoxia). The collected aortas were washed twice with ice-cold PBS and then immediately placed in liquid nitrogen. Total RNAs were extracted from the aortas by using TRIzol reagent (Thermo Fisher Scientific, Waltham, MA, USA). RNA sequencing technology was provided by Shanghai OE Biotech Co., Ltd. (Shanghai, China). Principal component analysis (PCA) and hierarchical clustering were carried out using the R programming language. PCA was used on gene and sample requirement-filtered data to visualize differences between groups. Any gene with reads less than 10 and with samples less than 4 were removed. Differentially expressed genes (DEGs) were defined as up- or downregulated log2 (fold change) (FC) > 2 or log2 FC < −2 and *p* < 0.05. KEGG enrichment analysis of different cluster genes was implemented using clusterProfiler R package, and the cutoff for *p* value was set at 0.05.

### 2.5. Elastic Van Gieson Staining

The suprarenal abdominal aortic tissues were harvested, fixed in 4% paraformaldehyde in PBS, and embedded in paraffin for further analysis. Tissue sections with 5 *μ*m thickness were prepared and subsequently stained with elastic Van Gieson (EVG) according to the manufacturer's instructions (Abcam, Cambridge, MA). The degradation score of elastin was calculated as previously described [14].

### 2.6. Gelatin Zymography

To detect the activity of MMP2 and MMP9, 10% acrylamide-SDS gel containing 0.1% gelatin was accustomed to separate proteins extracted from mice or cell culture medium at 4°C. Then, the proteins were denatured with washing buffer for 1 hour. After 36 hours of incubation in developing buffer, the proteins were stained with Coomassie Brilliant Blue. The proteins were then destained till clear bands appeared on the blue background. MMP activities were quantified by Image-Pro Plus software (Media Cybernetics, Silver Spring, USA).

### 2.7. Transmission Electron Microscopy

The collected suprarenal abdominal aortic tissues were mixed with 2.5% gluteraldehyde. After rising with 0.1 M sodium cacodylate, tissues were postfixed with 1% osmium tetroxide for 1 h. The tissues were cut into sections with 80 nm thickness and then dehydrated in ethanol solutions and embedded in Epon mixture. The sections were stained by uranyl acetate (Ieda Chemicals, Tokyo, Japan) and lead citrate (Sigma-Aldrich, St. Louis, MO, USA) and analyzed by transmission electron microscopy (TEM, Hitachi, Tokyo, Japan).

### 2.8. Immunohistochemical Staining

The suprarenal abdominal aortic tissues were fixed with 4% paraformaldehyde, embedded in paraffin, and cut into 4 *μ*m thickness sections. Sections were incubated with primary antibodies against MMP2 (final dilution 1 : 100 *v*/*v*, Abcam), MMP9 (1 : 100 *v*/*v*, GeneTex, Irvine, CA, USA), and *α*-SMA (1 : 50 *v*/*v*, Abcam) and caspase-3 (cleaved Asp175, 1 : 50 *v*/*v*, GeneTex) at 4°C overnight. Secondary antibodies with dilution of 1 : 500 *v*/*v* (Abcam) were used to incubate sections for 1 h at room temperature. Images were taken and the integration optical density value of MMP2-, MMP9-, and *α*-SMA-positive staining was measured by Image-Pro Plus software (Media Cybernetics, Silver Spring, USA).

### 2.9. TUNEL Assay

TUNEL staining was conducted using POD, an in situ cell death detection kit (Roche, Basel, Switzerland) according to the manufacturer's instructions. DAPI was used to stain nuclei which could show the total number of cells. Image-Pro Plus software (Media Cybernetics, Silver Spring, USA) was performed to calculate the percentage of TUNEL-positive nuclei in section.

### 2.10. ROS Detection

After washing with PBS for twice, aortic tissue section with 10 *μ*m thickness was embedded in Tissue-Tek OOCT compound (Miles Inc., Elkhart, IN, USA). The sections were then stained by 5 *μ*M dihydroethidium (DHE) fluorescent dye (Qcbio Science & Technologies Co., Ltd., Shanghai, China) for 60 min at 37°C. DHE staining images were taken under microscope (Olympus, Tokyo, Japan) with 518 nm excitation/610 nm emission filters.

### 2.11. Primary VSMC Isolation and Culture

The primary vascular smooth muscle cells (VSMCs) were isolated from mouse aortas as previously described [15]. The primary VSMCs were cultured in DMEM (Invitrogen, Carlsbad, CA, USA) with 20% fetal bovine serum (Gibco, Grand Island, NY, USA) at 37°C in a humidified atmosphere with 5% CO_2_. Primary VSMCs at passages 4 to 6 were used in the current study. Cells were serum-starved in DMEM for 24 h and then treated by 1 *μ*mol/L Ang II for 24 h. For inhibiting the CamKII signal, 40 nM CamKII-IN-1 (MedChemExpress, Monmouth Junction, NJ, USA) were added in cell for 1 h before Ang II treatment.

### 2.12. In Vitro CIH Model

After Ang II intervention, primary VSMCs in the CIH group were maintained in a chamber (model PH-1A, PUHE biotechnology Company Ltd., Wuxi, China) with 5% CO_2_ at 37°C. The O_2_ levels of the chamber were shifted from 21% for 5 min to 1% for 10 min. After 64 cycles, the cells were collected for use in the following experiments.

### 2.13. mtROS Detection

mtROS was detected with MitoSOX Red (Invitrogen, Carlsbad, CA, USA). Cells were incubated with 5 *μ*mol/L MitoSOX Red at 37°C for 30 min in the dark and then washed twice with PBS for 10 min each time. Then, cells were counterstained with DAPI. Fluorescent staining images were acquired employing a confocal microscope (NikonC2, Nikon, Tokyo, Japan).

### 2.14. Flow Cytometry

After Ang II and CIH treatment, primary VSMCs were collected and adjusted to the single-cell suspension with a concentration of 1 × 10^6^/mL. The cell suspension was then stained by using the Annexin V-FITC/PE Apoptosis Assay Kit (BD Biosciences, San Jose, CA, USA). Apoptotic cells were recognized and calculated by the flow cytometry (FACS Calibur, Becton Dickson, San Jose, CA).

### 2.15. qRT-PCR

After Ang II and CIH treatment, primary VSMCs were extracted by TRIzol reagent (Thermo Fisher Scientific) and the purity of extracts was verified by testing absorbance at 260 and 280 nm. The mRNAs extracted was reverse-transcribed to cDNA by using PrimeScript™ RT reagent Kit (TaKaRa Biotechnology, Dalian, China). Quantitative PCR was performed by using TB Green Fast qPCR Mix (TaKaRa). The primer sequences used in the qPCR procedure were listed in Table 1. GAPDH was used as the internal control. All primers were verified for specificity using Blast Search (http://www.ncbi.nlm.nih.gov/entrez/BLAST/).

### 2.16. Western Blot

After Ang II and CIH treatment, the whole proteins in primary VSMCs were isolated by lysing cells with radioimmunoprecipitation assay (Beyotime, Shanghai, China). A equal amount of protein extracts was subjected to 12% SDS-PAGE and transferred onto nitrocellulose membranes (Bio-Rad, Hercules, CA, USA). The following primary antibodies were used to probe targeted proteins: anti-MMP9 (final volume 1 : 500 *v*/*v*, GeneTex), anti-MCP1 (1 : 1000 *v*/*v*, GeneTex), anti-GAPDH (1 : 5000 *v*/*v*, GeneTex), anti-Bax (1 : 500 *v*/*v*, GeneTex), anti-Bcl-2 (1 : 500 *v*/*v*, GeneTex), anti-caspase-3 (cleaved Asp175, 1 : 500 *v*/*v*, GeneTex), anti-p38 (1 : 500 *v*/*v*, GeneTex), anti-MMP2 (1 : 500 *v*/*v*, Abcam), anti-p-CaMKII (phospho T286, 1 : 1000 *v*/*v*, Abcam, Cambridge, MA), anti-CaMKII (1 : 1000 *v*/*v*, Abcam), anti-p-p38 (phospho T180, 1 : 1000 *v*/*v*, Abcam), anti-Jnk1/2/3 (phospho T183+T183+T221, 1 : 1000 *v*/*v*, Abcam), and anti-Jnk1/2/3 (1 : 1000 *v*/*v*, Abcam). After incubation with secondary antibodies (1 : 2000 *v*/*v*, Abcam) at room temperature for 1 h, the band was semiquantified by Image-Pro Plus software (Media Cybernetics, Silver Spring, USA).

### 2.17. Statistics

The statistical analysis of this study was performed by using SPSS 19.0 statistical software (SPSS Inc., Chicago, IL, USA). The normal distribution of continuous variables was evaluated using the Shapiro-Wilks test. Data presented as mean ± SD or median and interquartile ranges. Categorical variables were presented as frequencies and percentages. The survival rate between groups was tested by the Kaplan–Meier survival analysis. Chi-square test was for the incidence of aneurysm. According to continuous variables with normal distribution or not, a significant difference between three or more was analyzed by one-way ANOVA or Kruska-Wallis test. In one-way ANOVA for post hoc multiple comparison, the Student-Newman-Keuls (SNK) test was used. Nemenyi test was performed for the multiple comparisons in the Kruskal-Wallis test. Based on continuous variables with normal distribution or not, a significant difference between two groups was analyzed by *t*-test or Mann–Whitney *U* tests. A *p* value less than 0.05 was defined as a significant difference.

## 3. Results

### 3.1. CIH Exposure Increases AAA Incidence

**Figure 1:**
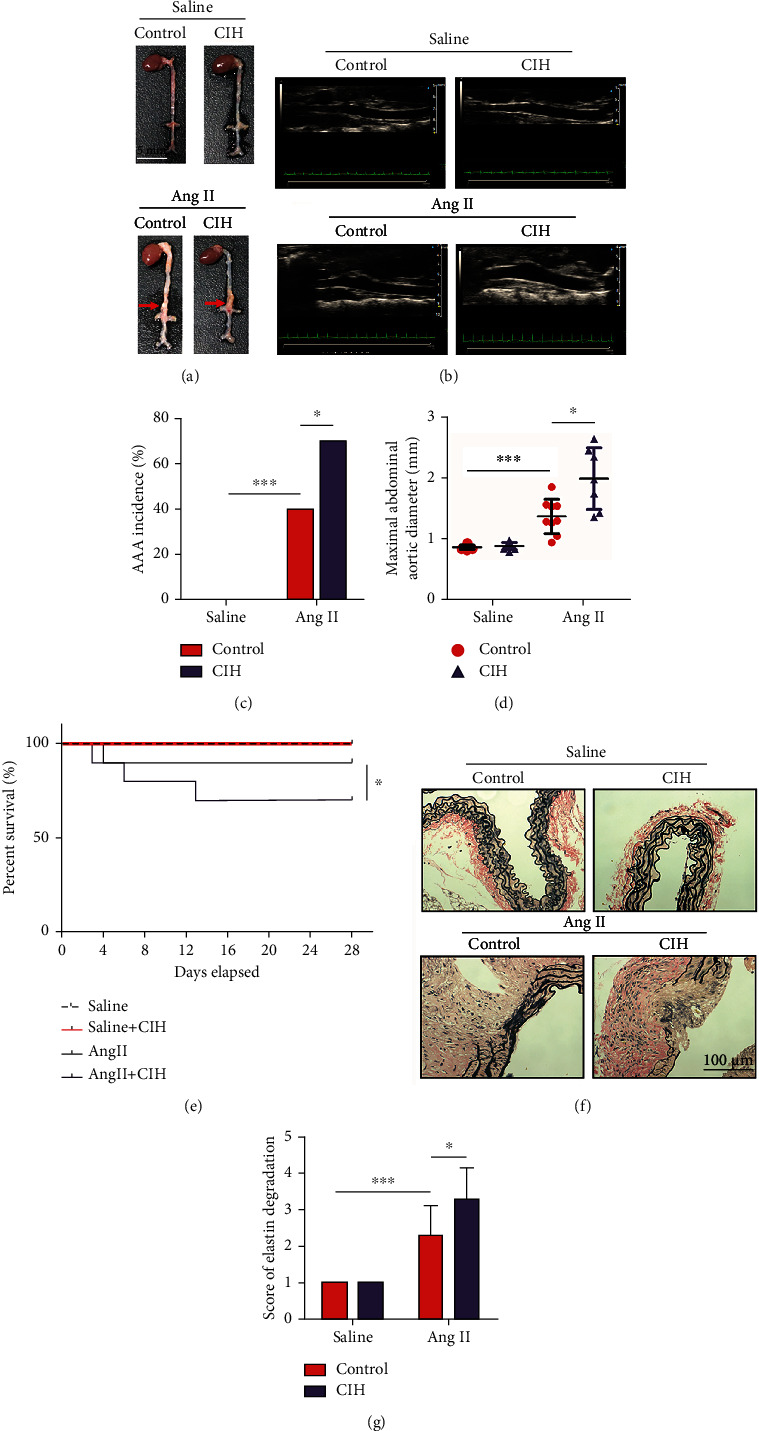
CIH exposure increases AAA incidence. An animal model of AAA was established by infusion Apoe^−/−^ mice with Ang II. The mice in the control group were infused with the same volume of saline. Mice were then exposed to CIH condition for 4 weeks. (a) Representative image of the whole aortae. (b) Representative ultrasound images. (c) The incidence of AAA (*n* = 10). (d) Maximal abdominal aortic diameter (*n* = 7-10). (e) Survival rate of mice in each group (*n* = 10). (f) Representative image of elastic Van Gieson (EVG) staining. (g) Score of elastin degradation from EVG staining (*n* = 7-10). ^∗^*p* < 0.05, ^∗∗∗^*p* < 0.001.

Firstly, we established AAA animal model through infusion of Apoe^−/−^ mice with Ang II. As seen in Figure 1(a) and the ultrasound images in Figure 1(b), Ang II infusion in mice developed generalized aortic dilation whereas saline did not induce any AAA in both the control and CIH groups. CIH seemed to induce a larger aortic dilation as compared with its negative control in Ang II-infused group. The incidence of AAA in the Ang II+CIH group was 70% (7/10) which is higher than the 40% (4/10) in the Ang II group (*p* < 0.05, Figure 1(c)). Also, the maximal abdominal aortic diameter of mice treated by Ang II+CIH was higher than that in the Ang II group (*p* < 0.05, Figure 1(d)). No mice died in the saline-infused groups. One mouse (10%) died from aortic rupture in the Ang II group, whereas 3 mice (33.3%) died in Ang II+CIH group (*p* < 0.05, Figure 1(e)). In addition, CIH significantly increased the elastin degradation score in Ang II-infused mice (*p* < 0.05, Figures 1(f) and 1(g)).

### 3.2. CIH Upregulates MMP Expression and Activity In Vivo

**Figure 2:**
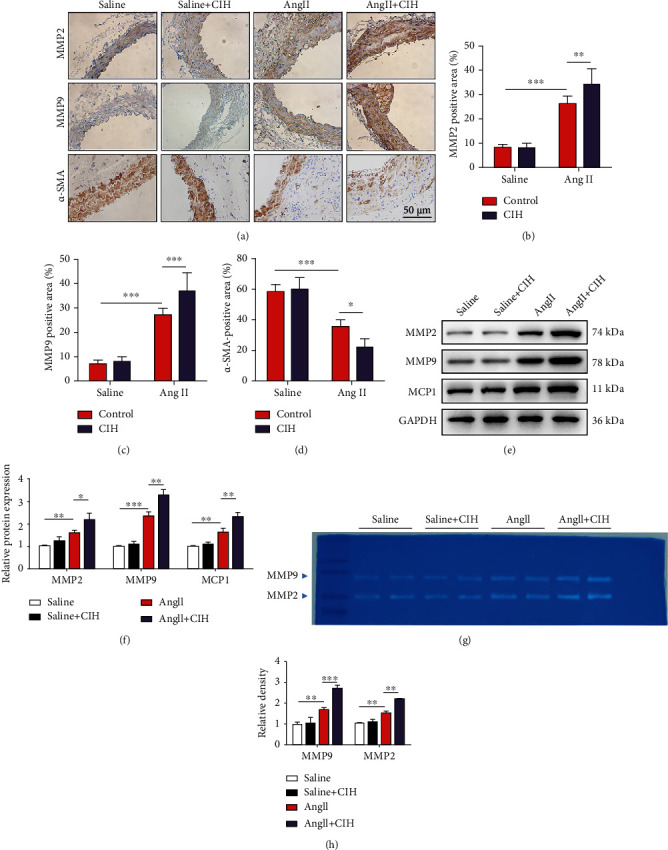
CIH upregulates AAA-related expression of MMP protein *in vivo*. An animal model of AAA was established by infusion Apoe^−/−^ mice with Ang II. The mice in the control group were infused with the same volume of saline. Mice were then exposed to CIH condition for 4 weeks. (a) Representative image of immunohistochemical. Quantification of (b) MMP2-, (c) MMP9-, and (d) *α*-SMA-positive area from immunohistochemical (*n* = 6). (e) Representative image of Western blot. (f) Quantification of MMP2, MMP9, and MCP1 protein expressions (*n* = 6). (g) MMP2 and MMP9 activities by zymography. (h) Quantification of MMP2 and MMP9 activities (*n* = 6). ^∗^*p* < 0.05, ^∗∗^*p* < 0.01, and ^∗∗∗^*p* < 0.001.

We next assessed the effects of CIH on the expression of MMP protein, which participates in extracellular matrix (ECM) remodeling and aneurysm formation. Immunohistochemical results in Figures 2(a)–2(c) show that the aortas from Ang II-infused mice exhibited a significant increase in the expression of MMP2 and MMP9 (*p* < 0.001). CIH further increased the MMP2 and MMP9 expressions in the aortas (*p* < 0.01 and *p* < 0.001). However, *α*-SMA, a marker of contractile VSMCs was lowly expressed in Ang II-infused mice (*p* < 0.001, Figures 2(a) and 2(d)). CIH exposure further reduced the expression of *α*-SMA (*p* < 0.05). Western blot results confirmed the promoting effects of CIH on MMP2 (*p* < 0.01) and MMP9 (*p* < 0.05) as well as the expression of MCP1 (*p* < 0.05, Figures 2(e) and 2(f)). Consistent with the increase in MMP expression, to determine whether altered MMP levels translate into proteolytic activity, MMP activity in aortic homogenates from each group was evaluated by zymography. The result revealed that Ang II-infusion in the Ang II group significantly increased MMP2 and MMP9 activities by zymography (*p* < 0.01). Additionally, CIH exposure markedly aggravated MMP2 and MMP9 activities (*p* < 0.01 and *p* < 0.001, Figures 2(g) and 2(h)).

### 3.3. CIH Induces ROS Generation and the Loss of VSMCs In Vivo

**Figure 3:**
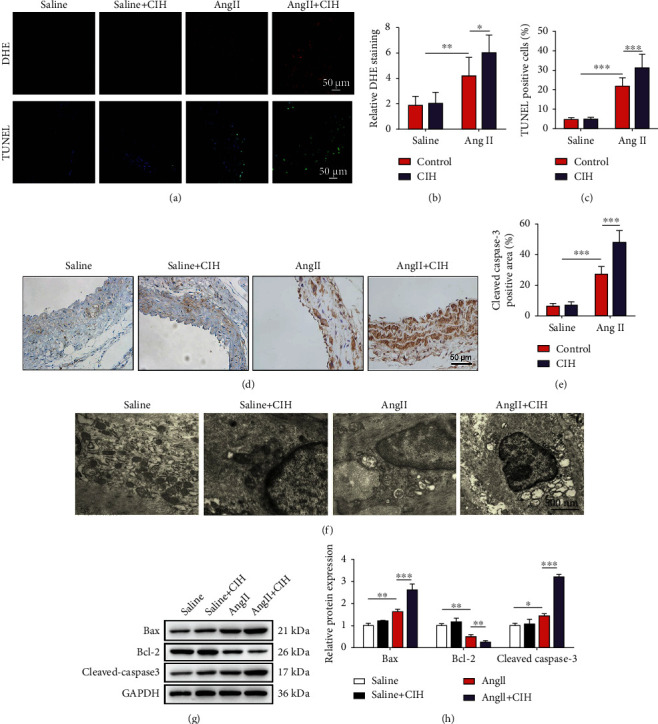
CIH induces ROS generation and the loss of VSMCs *in vivo*. An animal model of AAA was established by infusion Apoe^−/−^ mice with Ang II. The mice in the control group were infused with same volume of saline. Mice were then exposed to CIH condition for 4 weeks. (a) Representative image of DHE and TUNEL staining. (b) Quantification result of DHE staining (*n* = 6). (c) Quantification result of TNUEL staining (*n* = 6). (d) Representative image of immunohistochemical. (e) Quantification of cleaved caspase-3-positive area from immunohistochemical (*n* = 6). (f) TEM images (30000×) for the mitochondria of mouse aortic VSMCs. (g) Representative image of Western blot. (h) Quantification of Bax, Bcl-2, and cleaved caspase-3 protein expression (*n* = 6). ^∗^*p* < 0.05, ^∗∗^*p* < 0.01, and ^∗∗∗^*p* < 0.001.

ROS plays a key role in the regulation of MMP and VSMC loss. We therefore used DHE probe to detect oxidative stress and ROS generation in the aortic tissue. Immunohistochemical staining results in Figures 3(a) and 3(b) showed that CIH exposure markedly aggravated ROS activity, which was substantially induced by Ang II infusion (*p* < 0.05). TUNEL staining results indicated that CIH increased TUNEL-positive number in the aortic media of Ang II-infused mice (*p* < 0.001, Figures 3(a) and 3(c)). Cleaved caspase-3 immunostaining indicated increased apoptosis in the aortic media from the Ang II+CIH group than the Ang II group (*p* < 0.001, Figures 3(d) and 3(e)). TEM images in Figure 3(f) show that the mitochondria of mouse aortic VSMCs in the Ang II-infused group was remarkably damaged by CIH. To be specific, the loss of mitochondrial integrity, degradation of mitochondrial cristae, and swelling of the mitochondria were obvious in the Ang II+CIH group. Additionally, CIH exposure significantly upregulated the Bax and cleaved caspase-3 expressions (both *p* < 0.01) while downregulating the Bcl-2 expression (*p* < 0.05, Figures 3(g) and 3(h)). Overall, these results demonstrate that CIH exposure induced ROS generation and promoted exaggerated VSMC apoptosis.

### 3.4. CIH Regulates CaMKII-Dependent MAPK Signaling

**Figure 4:**
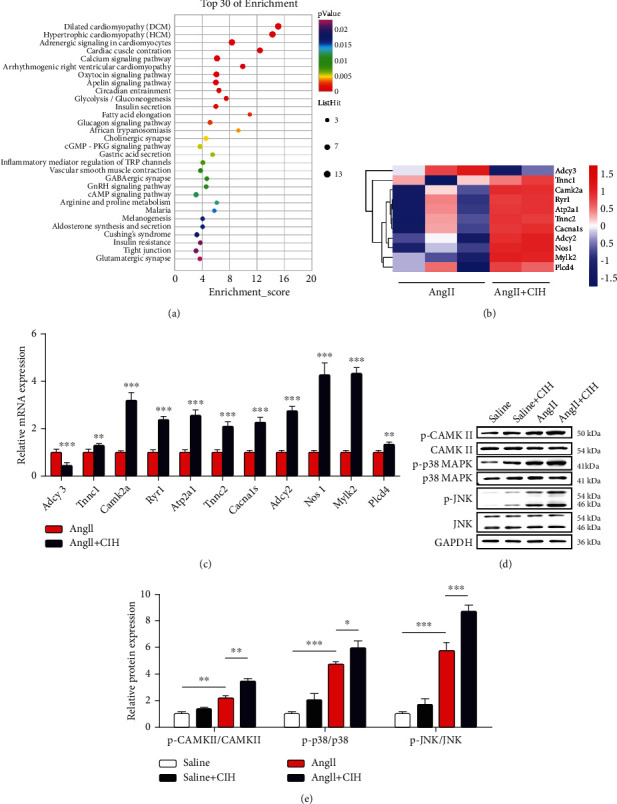
CIH regulates CaMKII-dependent MAPK signaling. (a) Apoe^−/−^ mice infused with Ang II and then exposed to CIH or normal conditions. Aortas tissues were collected for RNA sequencing and KEGG analysis. Top 30 of enriched signaling in aortas tissues were shown. (b) Hierarchical clustering and heat map showing the enrichment of the calcium signaling pathway genes in aorta tissues pretreated with normoxia or CIH. Red and blue indicate up- and downregulation, respectively. (c) qRT-PCR analysis of the mRNA expression levels of 11 calcium-related genes in aorta tissues treated with normoxia or CIH (*n* = 4). (d) Representative image of Western blot. (e) Quantification of CaMKII, p38, and Jnk from Western blot (*n* = 6). ^∗^*p* < 0.05, ^∗∗^*p* < 0.01, and ^∗∗∗^*p* < 0.001.

To explore the underlying mechanisms of which CIH aggravated Ang II-induced AAA, RNA sequencing of mouse aortas was performed. A total of 201 upregulated and 31 downregulate mRNAs in the Ang II+CIH group were found as relative to the Ang II group (fold change > 2, *p* < 0.05) (Supplemental Figure 1). Notably, top 30 of enriched signaling from KEGG analysis were shown in Figure 4(a), from which we found some pathways related to cardiovascular regulation and vascular remodeling, including the calcium signaling pathway. Through RNA sequencing and KEGG analysis, calcium channel has been considered a key downstream signaling of CIH, as the calcium-related genes like Tnnc1, Camk2a, Ryr1, and Atp2a1 were abnormally expressed (Figure 4(b)). We next confirmed several representative genes by performing qRT-PCR analysis. As compared with the Ang II group, Adcy3 was downregulated (*p* < 0.001), while Tnnc1, Camk2a, Ryr1, Atp2a1, Tnnc2, Cacna1s, Adcy2, Nos1, Mylk2, and Plcd4 were upregulated (*p* < 0.01 or *p* < 0.001) in the Ang II+CIH group (Figure 4(c)). Western blot results confirmed the promoting effects of CIH on CaMKII activation (*p* < 0.01, Figures 4(d) and 4(e)). Besides, CIH significantly induced the phosphorylation of p38 and Jnk (*p* < 0.05 and *p* < 0.001).

### 3.5. CIH Upregulates MMP Expression and mtROS Generation In Vitro

**Figure 5:**
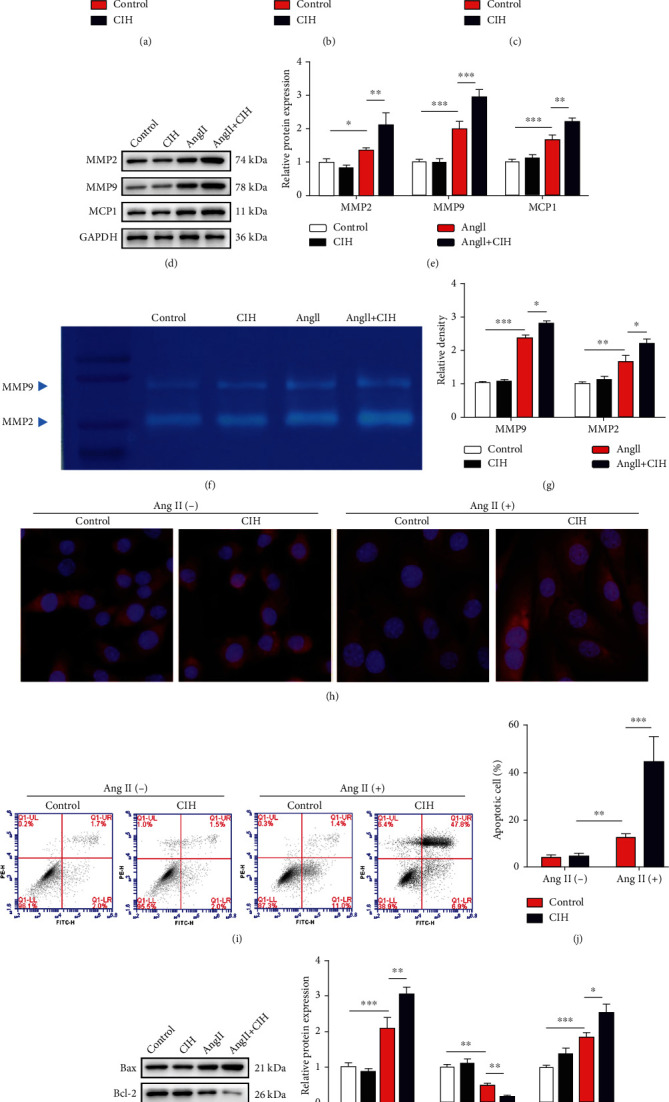
CIH upregulates MMP expression and mtROS generation *in vitro*. The isolated VSMCs from mouse aortas tissues were stimulated with Ang II combined with CIH. (a) The mRNA levels of MMP2, (b) MMP9, and (c) MCP1 were measured by qRT-PCR (*n* = 3). (d) Representative image of Western blot. (e) Quantification of MMP2, MMP9, and MCP1 protein expressions (*n* = 3). (f) MMP2 and MMP9 activities by zymography. (g) Quantification of MMP2 and MMP9 activities (*n* = 3). (h) Representative images showing mtROS levels (*n* = 3). (i) Representative image of flow cytometry for testing apoptosis. (j) Quantification of apoptotic cells from flow cytometry (*n* = 3). (k) Representative image of Western blot. (l) Quantification of Bax, Bcl-2, and cleaved caspase-3 expressions (*n* = 3). ^∗^*p* < 0.05, ^∗∗^*p* < 0.01, and ^∗∗∗^*p* < 0.001.

To confirm the effects of CIH on aortic VSMC damage, VSMCs were isolated and stimulated with Ang II combined with CIH. The mRNA levels of MMP2 (*p* < 0.01), MMP9 (*p* < 0.001), and MCP1 (*p* < 0.001) were significantly upregulated by CIH in Ang II-treated cell (Figures 5(a)–5(c)). Consistently, protein levels of MMP2 (*p* < 0.01), MMP9 (*p* < 0.05), and MCP1 (*p* < 0.05) were elevated by CIH (Figures 5(d) and 5(e)). Zymography results revealed that Ang II-infusion in the Ang II group significantly increased the MMP2 and MMP9 activities (*p* < 0.01 and *p* < 0.001, Figures 5(f) and 5(g)). Additionally, CIH exposure markedly aggravated MMP2 and MMP9 activities (both *p* < 0.05, Figures 5(f) and 5(g)). The fluorescent probe MitoSOX Red was used to detect changes in mtROS levels. CIH significantly elevated mtROS levels (Figure 5(h)). The apoptosis in VSMCs was induced by Ang II and further enhanced by CIH (*p* < 0.001, Figures 5(i) and 5(j)). The protein levels of Bax and cleaved caspase-3 were upregulated while Bcl-2 was downregulated by CIH in VSMCs (all *p* < 0.05, Figures 5(k) and 5(l)).

### 3.6. CIH Damages VSMCs through CaMKII-Dependent MAPK Signaling

**Figure 6:**
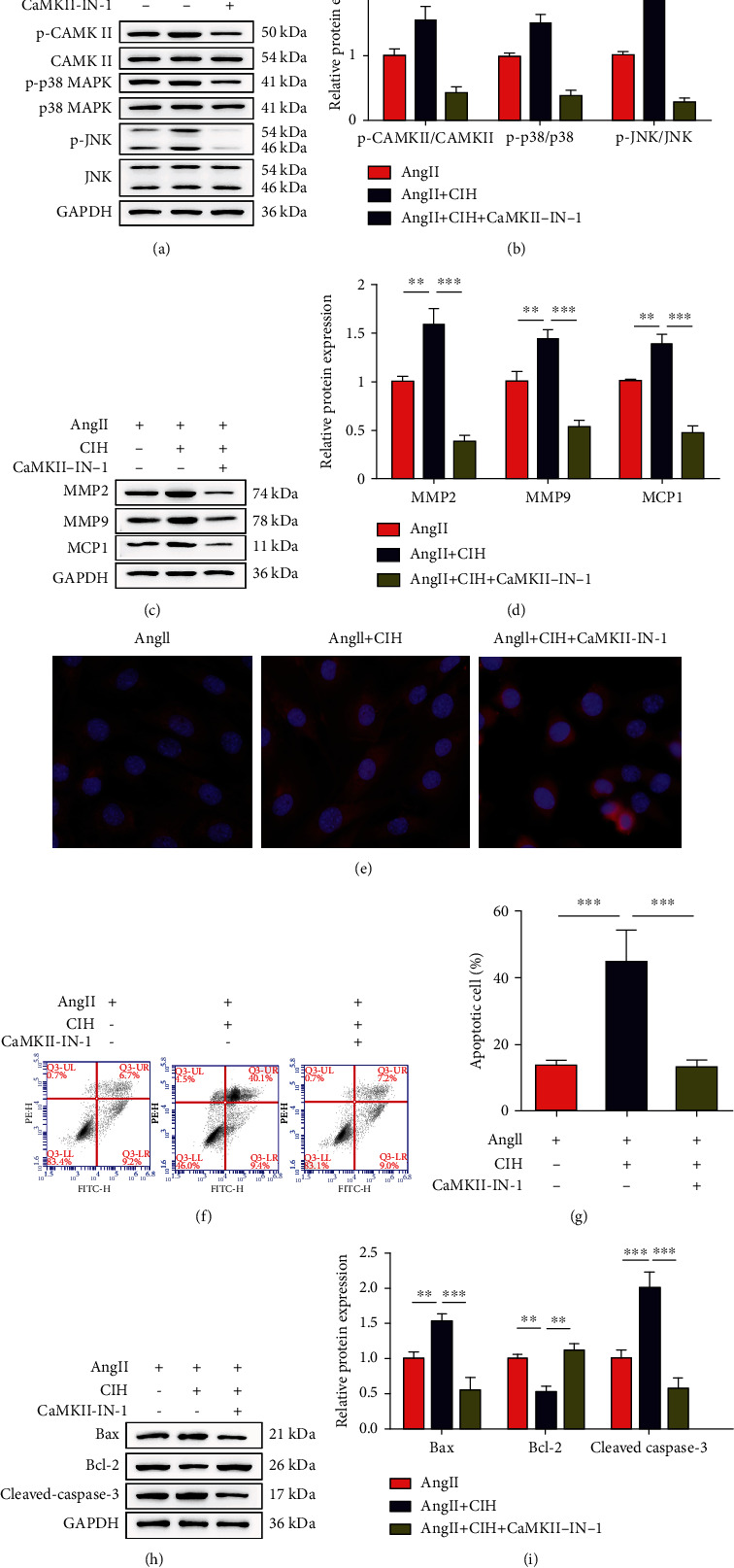
CIH damages VSMCs through CaMKII-dependent MAPK signaling. The isolated VSMCs from mouse aortas tissues were stimulated with Ang II combined with CIH. An inhibitor of calcium channel (CaMKII-IN-1) was used to treat VSMCs. (a) Representative image of Western blot. (b) Quantification of CaMKII, p38, and Jnk from Western blot (*n* = 3). (c) Representative image of Western blot. (d) Quantification of MMP2, MMP9, and MCP1 from Western blot (*n* = 3). (e) Representative images showing mtROS levels (*n* = 3). (f) Representative image of flow cytometry for testing apoptosis. (g) Quantification of apoptotic cells from flow cytometry (*n* = 3). (h) Representative image of Western blot. (i) Quantification of Bax, Bcl-2, and cleaved caspase-3 expressions (*n* = 3). ^∗∗^*p* < 0.01, ^∗∗∗^*p* < 0.001.

At last, CaMKII-IN-1, an inhibitor of calcium channel, was used to treat VSMCs. As results shown in Figures 6(a) and 6(b), the phosphorylation of CaMKII, p38, and Jnk were all inhibited by CaMKII-IN-1 (all *p* < 0.01). Adding with CaMKII-IN-1 significantly reduced both the basal and Ang II-induced expressions of MMP2, MMP9, and MCP1 (all *p* < 0.001, Figures 6(c) and 6(d)). The MitoSOX Red staining results indicated that CIH significantly elevated mtROS levels while CaMKII-IN-1 significantly reduced the levels of mtROS (Figure 6(e)). The apoptosis of VSMCs induced by Ang II was totally eliminated by CaMKII-IN-1 (*p* < 0.001, Figures 6(f) and 6(g)). Moreover, the upregulation of Bax and cleaved caspase-3 (both *p* < 0.001), as well as the downregulation of Bcl-2 (*p* < 0.01) induced by Ang II, were eliminated by CaMKII-IN-1 (Figures 6(h) and 6(i)).

## 4. Discussion

Previous studies have shown that OSA is related with the pathogenesis and progression of AAA [7, 8]. OSA is highly prevalent in patients with AAA, and CIH is considered to be the most important risk factor of AAA [7, 16]. Results of the current study confirmed the pathogenic role of CIH in AAA, as CIH exposure significantly enhanced AAA incidence and aortic diameter and reduced survival rate of mice with AAA. Additionally, CIH aggravated Ang II-induced elastin degradation and MMP expression. CIH induced the generation of ROS and thus promoted VSMC apoptosis. CaMKII-dependent p38/JNK signaling has been found to be a key underlying mechanism of CIH. And inhibition of CaMKII channel significantly attenuated the effects of CIH on VSMC apoptosis and MMP expression.

The pathogenesis of AAA has been identified as a complex remodeling process marked by the degradation of ECM [17]. Collagen and elastin are the most important ECM proteins which are responsible for maintaining the tensile strength of blood vessels and preventing aortic dilation and aneurysm rupture [18]. In the present study, elastin fragmentation was found to be promoted by CIH in mice. Additionally, MMP2 and MMP9 are two mediators of ECM degradation, secreted from monocytes, macrophages, and dysfunctional VSMCs [19]. The elevated MMP activity-induced vascular middle layer degradation is critical for the pathogenesis of AAA. In this study, we showed that CIH exposure significantly increased the activity and expression level of MMP2 and MMP9. All these evidenced the promoting effects of CIH on ECM degradation, which may further lead to AAA initiation.

Healthy VSMCs within the aortic media are important for normal vessel function. VSMCs are the main component of aortic vascular wall. VSMCs alternately form vascular media with elastic fiber layer and regulate vascular tension through systolic and diastolic functions. The change of smooth muscle structure is the main pathological basis of AAA, and the loss of aortic VSMCs is a hallmark of human AAA [20]. ROS generation and apoptosis of VSMCs can initiate aortic inflammation, which promote the initiation of AAA [21]. Previous studies have revealed that OSA increased ROS level and induced oxidative stress through CIH [22, 23], which led to OSA-related aortic diseases, like thoracic aortic aneurysm and AAA [24]. In the current study, significant ROS generation and VSMC apoptosis were found in mice. Intracellular ROS is mainly produced by mitochondria. Mitochondria-derived ROS (mtROS) plays an important role in causing apoptosis via the intrinsic pathway [25]. In this study, mtROS levels significantly increased by Ang II, which was further increased by CIH using the fluorescent probe MitoSOX Red. Additionally, the loss of mitochondrial integrity, degradation of mitochondrial cristae, and swelling of the mitochondria were obvious in the Ang II+CIH group. Mitochondrial apoptotic pathway, also referring to intrinsic cell death pathway, is a process during cell death induced by intracellular stressors such as DNA damage, serum deprivation, and oxidative stress and is negatively regulated by Bcl-2 protein and positively regulated by Bax and Bak [26]. In this study, as expected, we found that CIH exposure significantly upregulated the expression of Bax and cleaved caspase-3 while downregulating Bcl-2 expression. Previous studies have suggested that mitochondrial apoptosis pathway plays a vital role in the pathogenetic mechanism in cardiovascular diseases including aortic aneurysm and dissection [27, 28]. Therefore, we hypothesized that the CIH treatment may promote excessive VSMC apoptosis under the status of mitochondrial death pathway in the aneurysmal aorta. Additionally, CIH was found to upregulate the expression of MCP1, a key inflammatory molecule implicated in AAA [29]. mtROS generation and VSMC apoptosis will promote aortic inflammation and thus lead to aortic aneurysm [21]. This finding further confirmed the promoting effects of CIH on the initiation of AAA through inducing VSMCs loss.

To reveal the underlying mechanism of which CIH promote the initiation of AAA, RNA sequencing and KEGG analyses were performed. Calcium signaling pathway is one of the top 30 enriched signaling in mouse aortas after Ang II and CIH treatment. CaMKII is a serine/threonine-specific phosphokinase that has an emerging role in cardiac remodeling [30]. Besides, CaMKII signaling triggers mechanisms in the heart and thus is involved in the pathogenesis of heart failure [31] and arrhythmia [32]. CaMKII is expressed in VSMCs, and nuclear CaMKII translocates to the cytoplasm in response to Ang II for inducing arterial hypertension [33]. Besides, CaMKII is critical in controlling the proliferation [34], hypertrophy [35], and migration [36] of VSMCs, as well as the contractility of aorta [37]. In this study, CaMKII was found to be phosphorylated by Ang II and CIH sharply accelerated CaMKII activation. Adding with an inhibitor of CaMKII (CaMKII-IN-1) could significantly eliminate the promoting effects of CIH on ECM degeneration and VSMC loss. These findings suggested CaMKII as one of the downstream signaling of CIH in inducing AAA.

MAPK is a key pathway involved in regulating cell survival, proliferation, apoptosis and stress response [38]. Activation of the MAPK pathway is involved in the pathogenesis of AAA [39, 40]. The three subfamily members of the MAPK pathway, ERK, Jnk, and p38, are highly expressed in the human and mouse AAA tissues [41]. And they regulate a wide range of pathological processes to promote ECM degradation and the release of proinflammatory cytokines, which ultimately contribute to AAA formation [41]. Previous studies have revealed that calcium channel could affect the transduction of MAPK signal [42, 43]. It was also confirmed in this study, as CaMKII-IN-1 significantly inhibited the activation of both p38 and Jnk in VSMCs treated with Ang II and CIH. It seems that CIH promote the initiation of AAA possibly through CaMKII-dependent MAPK signaling [27].

## 5. Conclusions

To conclude, a reliable animal model of AAA was established by infusing Apoe^−/−^ mice with Ang II and exposure in CIH. The model contains phenotype of both AAA and OSA. We confirmed the emerging role of OSA in the initiation of AAA, as CIH remarkably induced ECM generation and the loss of VSMCs. OSA promoted the initiation of AAA possibly through regulating CaMKII-dependent MAPK signaling.

## Figures and Tables

**Table 1:** The primer sequences used in the qRT-PCR analysis.

Primer	Accession no.	Sequences
Adcy3	NM_138305	5′-CTCGCTTTATGCGGCTGAC-3′ (forward) 5′-TCATGGCGCTGCCTTTTGAA-3′ (reverse)
Tnnc1	NM_009393	5′-GCGGTAGAACAGTTGACAGAG-3′ (forward) 5′-CCAGCTCCTTGGTGCTGAT-3′ (reverse)
Camk2a	NM_177407	5′-TATCCGCATCACTCAGTACCTG-3′ (forward) 5′-GAAGTGGACGATCTGCCATTT-3′ (reverse)
Ryr1	NM_009109	5′-CGCACACAGTCGTATGTACCT-3′ (forward) 5′-TAATCCCACGTCAAAGGCCAA-3′ (reverse)
Atp2a1	NM_007504	5′-TGTTTGTCCTATTTCGGGGTG-3′ (forward) 5′-AATCCGCACAAGCAGGTCTTC-3′ (reverse)
Tnnc2	NM_009394	5′-GGGGACATCAGCGTTAAAGAG-3′ (forward) 5′-GCGTTCCTGTCAAAGATGCG-3′ (reverse)
Cacna1s	NM_014193	5′-TCAGCATCGTGGAATGGAAAC-3′ (forward) 5′-GTTCAGAGTGTTGTTGTCATCCT-3′ (reverse)
Adcy2	NM_153534	5′-GACTGGCTCTACGAGTCCTAC-3′ (forward) 5′-GGGCAGTGGGAACGGTTAT-3′ (reverse)
Nos1	NM_008712	5′-CTGGTGAAGGAACGGGTCAG-3′ (forward) 5′-CCGATCATTGACGGCGAGAAT-3′ (reverse)
Mylk2	NM_001081044	5′-GCGAGACAACAGACCTCGTC-3′ (forward) 5′-GGTGTCCCCTTGCACCTTAG-3′ (reverse)
Plcd4	NM_148937	5′-GAAGGTTATGAAGTGTCCGATGT-3′ (forward) 5′-AACTGCTTTGACAAGAGAATGGA-3′ (reverse)
MMP9	NM_013599	5′-CTGGACAGCCAGACACTAAAG-3′ (forward) 5′-CTCGCGGCAAGTCTTCAGAG-3′ (reverse)
MMP2	NM_008610	5′-CAAGTTCCCCGGCGATGTC-3′ (forward) 5′-TTCTGGTCAAGGTCACCTGTC-3′ (reverse)
MCP-1	NM_011333	5′-TAAAAACCTGGATCGGAACCAAA-3′ (forward) 5′-GCATTAGCTTCAGATTTACGGGT-3′ (reverse)
GAPDH	NM_008084	5′-AGGTCGGTGTGAACGGATTTG-3′ (forward) 5′-TGTAGACCATGTAGTTGAGGTCA-3′ (reverse)

## Data Availability

The data used to support the findings of this study are available from the corresponding author upon request.

## Supplementary Materials

Supplementary Materials Supplemental Figure 1: the change of mRNA expression pattern in aortas tissues.
