# Supplementary figures and images for: Chronic Intermittent Hypoxia Regulates CaMKII-Dependent MAPK Signaling to Promote the Initiation of Abdominal Aortic Aneurysm
Source: Oxid Med Cell Longev. 2021 Dec 21;2021:2502324. doi: 10.1155/2021/2502324 (PMC8714336; doi:10.1155/2021/2502324)

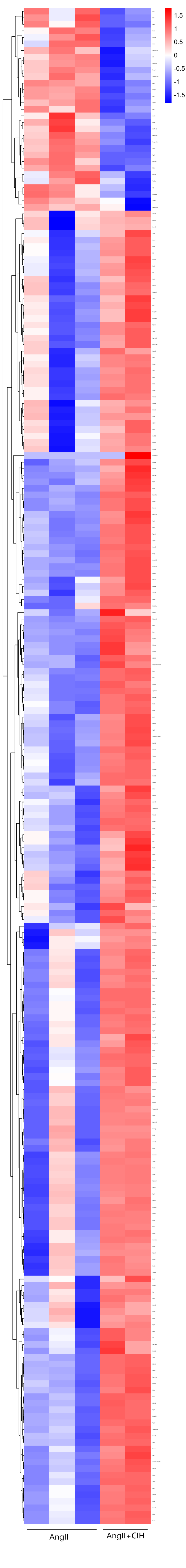

Supplement: Supplementary Materials — Supplemental Figure 1: the change of mRNA expression pattern in aortas tissues. [file 2502324.f1.pdf]
